# Examining the Role of Physician Characteristics in Web-Based Verified Primary Care Physician Reviews: Observational Study

**DOI:** 10.2196/51672

**Published:** 2024-07-29

**Authors:** Neil K R Sehgal, Benjamin Rader, John S Brownstein

**Affiliations:** 1 Department of Computer and Information Science University of Pennsylvania Philadelphia, PA United States; 2 Computational Epidemiology Group Boston Children's Hospital Boston, MA United States; 3 Department of Anesthesiology Critical Care and Pain Medicine Boston Children’s Hospital Boston, MA United States; 4 Department of Pediatrics Harvard Medical School Boston, MA United States

**Keywords:** patient review websites, patient online review, telemedicine, internet, online review, online reviews, rating, physician review, physician reviews, doctor review, doctor reviews

## Abstract

**Background:**

Doctor review websites have become increasingly popular as a source of information for patients looking to select a primary care provider. Zocdoc is one such platform that allows patients to not only rate and review their experiences with doctors but also directly schedule appointments. This study examines how several physician characteristics including gender, age, race, languages spoken in a physician’s office, education, and facial attractiveness impact the average numerical rating of primary care doctors on Zocdoc.

**Objective:**

The aim of this study was to investigate the association between physician characteristics and patient satisfaction ratings on Zocdoc.

**Methods:**

A data set of 1455 primary care doctor profiles across 30 cities was scraped from Zocdoc. The profiles contained information on the physician’s gender, education, and languages spoken in their office. Age, facial attractiveness, and race were imputed from profile pictures using commercial facial analysis software. Each doctor profile listed an average overall satisfaction rating, bedside manner rating, and wait time rating from verified patients. Descriptive statistics, the Wilcoxon rank sum test, and multivariate logistic regression were used to analyze the data.

**Results:**

The average overall rating on Zocdoc was highly positive, with older age, lower facial attractiveness, foreign degrees, allopathic degrees, and speaking more languages negatively associated with the average rating. However, the effect sizes of these factors were relatively small. For example, graduates of Latin American medical schools had a mean overall rating of 4.63 compared to a 4.77 rating for US graduates (*P*<.001), a difference roughly equivalent to a 2.8% decrease in appointments. On multivariate analysis, being Asian and having a doctor of osteopathic medicine degree were positively associated with higher overall ratings, while attending a South Asian medical school and speaking more European and Middle Eastern languages in the office were negatively associated with higher overall ratings.

**Conclusions:**

Overall, the findings suggest that age, facial attractiveness, education, and multilingualism do have some impact on web-based doctor reviews, but the numerical effect is small. Notably, bias may play out in many forms. For example, a physician's appearance or accent may impact a patient's trust, confidence, or satisfaction with their physician, which could in turn influence their take-up of preventative services and lead to either better or worse health outcomes. The study highlights the need for further research in how physician characteristics influence patient ratings of care.

## Introduction

There has been a growing interest in understanding the factors that may influence a patient's perception of their physician and how these perceptions might in turn impact quality of care. Research has shown that patients often rely on nonverbal cues, such as a physician's attire, to form their opinions about their health care provider [1-6]. With the advent of various web-based review platforms, such as Healthgrades and Zocdoc, patients have easy access to a wealth of information about their health care providers, as well as a relatively straightforward means to express their satisfaction. Research suggests that these web-based ratings do influence patients' choices of medical providers [7-9]. On the web-based physician review platform Zocdoc, a half-star improvement in ratings on a scale of 1-5 stars leads to a 10% increase in appointments [10,11].

Past research is mixed on the factors influencing these web-based reviews. Some research suggests that patient experience and clinical quality directly affect web-based ratings [12,13]. Other research suggests that there exists minimal correlation between the quality and value of care or peer-assessed performance with web-based ratings, and that nonphysician characteristics such as staff friendliness and appointment wait times are instead the key determinants [14-16].

What remains clear, however, is that many different factors play a role [17,18]. Web-based reviews for female physicians, for example, have been found to be more emotional and informal compared to those for male physicians [19]. Moreover, female physicians receive lower web-based ratings than their male counterparts on some platforms, though this trend is not consistent across all platforms [19-23]. Other research finds that patient-physician racial concordance is associated with higher scores on internal patient satisfaction surveys [24]. One study of a direct-to-consumer telemedicine platform shows that, on average, patients report higher rates of dissatisfaction with Black and Asian physicians compared to White physicians [25].

However, the influence of physician factors on physician ratings is likely not limited to gender and race alone. Despite the growing influence of web-based review sites, much remains unknown about the various factors that may influence physician ratings. For instance, many studies have examined the association between patient attractiveness and physician care practices [26-29]. However, to our knowledge, no study has examined how physician attractiveness impacts patient satisfaction in real-world medical settings.

Other factors such as school ranking and multilingualism are similarly understudied. Existing research finds little correlation between medical school ranking and performance scores [30]. In addition, language-concordant care is associated with increased patient satisfaction [31,32]. However, the roles of ranking and multilingualism have not been examined in web-based review settings.

In this paper, we aim to explore the role of various physician characteristics, including gender, age, race, number of languages spoken in a physician’s office, education, and facial attractiveness, in shaping patient perceptions of their physicians through ratings on a web-based doctor review site.

## Methods

We collected primary care doctor profile data from Zocdoc, a platform that allows patients to rate and review their experiences with doctors. Physician variables included facial attractiveness, race, age, gender, language, and education. Outcome variables included overall satisfaction, bedside manner, and wait time scores from patient reviews. Descriptive statistics, the Wilcoxon rank sum test, and multivariate logistic regression were used to analyze the data.

### Data Collection

We collected a data set of primary care doctor profiles from Zocdoc, a web-based platform that allows patients to search for and schedule telemedicine and in-person appointments with doctors, and also to rate and review their experiences. Notably, unlike many other sites that host physician reviews, Zocdoc only posts reviews from patients who have attended an appointment [33]. After appointments, patients receive emails from Zocdoc asking for feedback. Zocdoc is free for patients to use, and makes money by charging health care providers subscription or booking fees [34]. Health care providers may come from both independent practices and integrated networks. Patients can search for a physician using criteria such as condition, specialty, city, state, ZIP code, insurance carrier and plan, or a specific doctor's name. They can then filter by date, time of day, distance, gender, in-person or video consultations, hospital affiliation, and languages spoken by the physician.

Zocdoc does not maintain a public-centralized list of all providers on the platform. In order to systematically collect a large sample of providers, we used Zocdoc’s search engine to identify primary care doctors within 50 miles of each of the 30 largest cities in the United States [35]. Specifying ‟primary care doctor” in the search bar yields a variety of specialties, including internists, nurse practitioners, and physician assistants. We filtered these results specifically to profiles that listed their specialty as primary care doctor. We collected these profiles using a browser-based web scraping tool [36].

### Physician Variables

From each profile, we collected the listed gender, languages spoken in the physician’s office, type of medical school degree (allopathic or osteopathic), medical school institution, and downloaded the physician’s profile picture. All physicians were either graduates of allopathic medical schools, which grant doctor of medicine degrees, or osteopathic medical schools, which grant doctor of osteopathic medicine degrees. We determined the geographical location of each medical school (Africa, the Caribbean, East Asia, Europe, Latin American, the Middle East, South Asian, the mainland United States or Canada, other) by searching for each medical school on Google. The US medical schools were additionally coded for whether or not they were ranked in the top 30 of the US News & World Report’s 2023-2024 Medical School Research rankings [37]. For analysis purposes, we divided the number of languages spoken into buckets (1, 2, 3, and ≥4). We additionally broke down the number of languages into 7 categories: European, East and Southeast Asian, South Asian, Middle Eastern, African, Caribbean, and Creole (Table S1 in Multimedia Appendix 1).

Through Face++, a commercial facial analysis software commonly used to infer demographic factors, we imputed a facial attractiveness rating for each profile [38-42]. Face++’s estimation of facial attractiveness has been found to correlate well (*r*=0.72) with human raters [42]. We use a second commonly used commercial facial analysis software, Kairos, to infer race and age, which has been found to outperform Face++’s race and age models [43-46]. One study from 2019 found the mean absolute error for Kairos’ age model to be ±3.30 (SD 2.64) years when compared to human raters’ estimates as ground truth [43]. Accuracy for Kairos’ race model was 95.06% (95% CI 94.08%-95.93%) when compared to human raters [43]. Similar methods have been used to understand the diversity in hospital system faculty and medical editorial boards [47,48]. To examine potential nonlinearities and for interpretability purposes, we divided facial attractiveness and age into quartiles. For robustness, we compared Face++’s age prediction results with those of Kairos (Tables S2-S3 in Multimedia Appendices 2 and 3).

### Outcome Variables

Each doctor profile was associated with 3 average scores from patient reviews: overall satisfaction, bedside manner, and wait time. Each score is rated on a scale of 1-5 stars. We additionally collected the total number of reviews. Profiles with 0 reviews were removed from the sample.

### Statistical Analysis

The data are analyzed at the physician level. We used descriptive statistics to summarize the data and the Wilcoxon rank sum test to determine if there were significant differences in average review scores across gender, age quartile, facial attractiveness quartile, education, and number of languages spoken. Multivariable logistic regression was used to assess the association between these factors and average review scores. The outcome for the regression is a binary variable indicating whether or not the physician is above the 25th percentile of ratings to understand factors associated with very low ratings. All statistical analyses were conducted using R (version 4.1.2; The R Foundation).

### Ethical Considerations

This study used publicly available data posted for public use by the providers, and therefore, did not require institutional review board approval.

## Results

A total of 1521 primary care doctor profiles from Zocdoc were collected. Of these, 66 were missing an overall satisfaction rating and excluded, leaving a sample of 1455 primary care doctor profiles for analysis (Table 1). One profile lacked information on the medical school attended, one profile contained a profile picture from which facial attractiveness, age, and race could not be estimated, and one profile contained a profile picture for which only facial attractiveness could not be assessed. These 3 profiles were still included for analysis. Three cities—Houston, Chicago, and New York City—represented 49% of all primary care doctors in the sample (Table S4 in Multimedia Appendix 4). The majority of physicians were men (54%) with no nonbinary individuals represented in the study. The sample included speakers of 66 languages, with a mean of 1.73 languages spoken (Table S1 in Multimedia Appendix 1).

**Table 1:** Characteristics of Zocdoc primary care profiles (N=1455^a^).

Characteristics				
Overall satisfaction rating, median (IQR)				4.82 (4.67-4.93)
Wait time rating, median (IQR)				4.67 (4.45-4.83)
Bedside manner rating, median (IQR)				4.88 (4.75-4.98)
Number of reviews, median (IQR)				57 (19-230)
**Gender, n (%)**				
	Female		675 (46)	
	Male		780 (54)	
Age (in years), mean (SD)				31 (8)
**Race, n (%)**				
	Asian		598 (41)	
	Black		107 (7.4)	
	Hispanic		149 (10)	
	White		600 (41)	
Facial attractiveness, mean (SD)				56 (12)
Top 30 medical school, n (%)				106 (7.3)
**Degree, n (%)**				
	DO^b^		279 (19)	
	MD^c^		1176 (81)	
**Medical school location, n (%)**				
	Africa		28 (1.9)	
	Caribbean		141 (9.7)	
	East and Southeast Asia		40 (2.8)	
	Europe		68 (4.7)	
	Latin America		59 (4.1)	
	Middle East		38 (2.6)	
	Other		3 (0.2)	
	South Asia		180 (12)	
	United States or Canada		897 (62)	
**Number of languages spoken in office, n (%)**				
		1	858 (59)	
		2	326 (22)	
		3	165 (11)	
		≥4	106 (7.3)	
Number of European languages, mean (SD)				1.29 (0.60)
Number of South Asian languages, mean (SD)				0.26 (0.73)
Number of East or Southeast Asian languages, mean (SD)				0.07 (0.33)
Number of Middle Eastern languages, mean (SD)				0.06 (0.26)
Number of African languages, mean (SD)				0.0158 (0.1543)
Number of Creole languages, mean (SD)				0.0034 (0.0585)

^a^Bedside rating and wait time rating were missing from 35 profiles; age, race, attractiveness, and medical school were missing from 1-2 profiles.

^b^DO: doctor of osteopathic medicine.

^c^MD: doctor of medicine.

### Univariate Analysis

**Figure 1:**
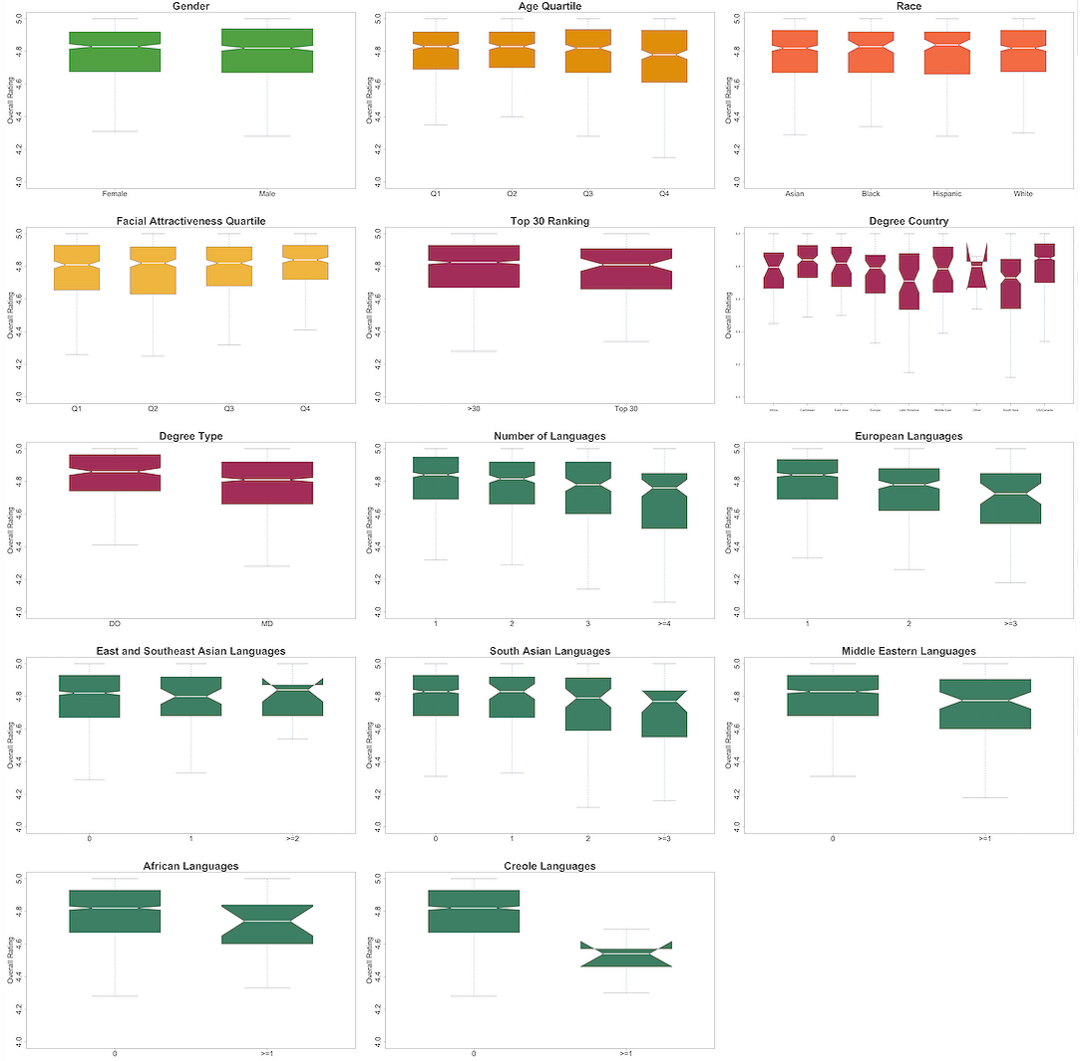
Overall primary care physician Zocdoc ratings by physician demographic factor. A higher resolution version of this figure is available in Multimedia Appendix 6.

Average overall ratings on Zocdoc are typically highly positive with a median of 4.82 (IQR 4.67-4.93). We find significant but small differences in mean overall rating by physician characteristics including age quartile, facial attractiveness quartile, degree country, degree type, and number of languages spoken (Figure 1, Table S5 in Multimedia Appendix 5). For example, the median overall rating for primary care doctors in the first quartile of age is 4.83 (IQR 4.69-4.92) but 4.78 (IQR 4.61-4.93) for doctors in the fourth quartile (Wilcoxon rank sum test, *P*=.02). We find similar results when using Face++’s age estimates (Table S2 in Multimedia Appendix 2). The median overall rating for primary care doctors in the first quartile of facial attractiveness is 4.81 (IQR 4.66-4.93) but 4.84 (IQR 4.72-4.93) for doctors in the fourth quartile (*P*=.03). Osteopathic physicians have a median overall rating of 4.86 (IQR 4.74-4.96) compared to 4.81 (IQR 4.66-4.92) for allopathic physicians (*P*<.001). The median overall rating for primary care doctors with a US or Canadian degree is 4.85 (IQR 4.70-4.94) compared to 4.79 (IQR 4.61-4.90) for those with a foreign degree (*P*<.001). However, not all foreign-educated physicians have significantly lower scores. For example, Caribbean-educated physicians have a median rating of 4.84 (IQR 4.73-4.93). Physicians educated in Latin America, however, have a median overall rating of 4.71 (IQR 4.54-4.88), a gap of 0.14 in median overall ratings compared with the US- or Canadian-educated physicians. Doctors with 1 language spoken in their office have a median overall rating of 4.84 (IQR 4.69-4.95) while doctors with ≥4 languages have a median overall rating of 4.76 (IQR 4.52-4.85) (*P*<.001). We observe no significant differential effects in overall ratings by gender across the other variables.

Average bedside manner rating is similarly highly positive with a median of 4.88 (IQR 4.75-4.98). We find significant but small differences by physician characteristics including age quartile, degree type, medical school location, and number of languages spoken (Table S5 in Multimedia Appendix 5). For example, the median bedside manner rating for primary care doctors in the first quartile of age is 4.89 (IQR 4.77-4.97) but 4.84 (IQR 4.70-4.96) for doctors in the fourth quartile (*P*<.001). Osteopathic physicians have a median bedside manner rating of 4.91 (IQR 4.81-5.00) compared to 4.87 (IQR 4.73-4.97) for allopathic physicians (*P*<.001). The median bedside manner rating for primary care doctors with a US or Canadian degree is 4.90 (IQR 4.77-5.00) compared to 4.85 (IQR 4.70-4.95) for those with a foreign degree (*P*<.001). Doctors with 1 language spoken in their office have a median bedside manner rating of 4.90 (IQR 4.77-5.00) while doctors with ≥4 languages have a median bedside manner rating of 4.81 (IQR 4.58-4.91; *P*<.001).

The average wait time rating is similarly highly positive with a median of 4.67 (IQR 4.45-4.83). We find significant but small differences by physician characteristics including age quartile, facial attractiveness quartile, degree type, medical school location, and number of languages spoken (Table S5 in Multimedia Appendix 5). For example, the median wait time rating for primary care doctors in the first quartile of age is 4.72 (IQR 4.50-4.86) but 4.62 (IQR 4.38-4.79) for doctors in the fourth quartile (*P*<.001). The median wait time rating for primary care doctors in the first quartile of facial attractiveness is 4.64 (IQR 4.40-4.81) but 4.71 (IQR 4.53-4.85) for doctors in the fourth quartile (*P*=.003). Osteopathic physicians have a median wait time rating of 4.74 (IQR 4.54-4.88) compared to 4.65 (IQR 4.42-4.82) for allopathic physicians (*P*<.001). The median wait time rating for primary care doctors with a US degree is 4.69 (IQR 4.50-4.84) compared to 4.62 (IQR 4.40-4.81) for those with a foreign degree (*P*=002). Doctors with 1 language spoken in their office have a mean wait time rating of 4.70 (IQR 4.50-4.85) while doctors who speak ≥4 languages have a median wait time rating of 4.46 (IQR 4.24-4.67; *P*<.001).

Having excluded 66 profiles with zero reviews, the vast majority of profiles have multiple reviews with a median of 57 (IQR 19-230). We find significant differences in the number of reviews by facial attractiveness quartile, number of languages spoken, and degree type. For example, the median number of reviews for doctors below the 25th percentile of facial attractiveness is 50, but 73 for doctors above the 75th percentile (*P*=.009). The median number of reviews for doctors with 1 language is 42, while doctors with ≥4 languages have a median number of reviews of 244 (*P*<.001). The median number of reviews for osteopathic physicians is 44 compared to 61 for allopathic physicians (*P*=.01). Linear regression analysis finds no association between average overall rating and number of reviews.

### Multivariate Analysis

On multivariate analysis (Table 2), being older, attending a South Asian medical school, and speaking more European and Middle Eastern languages are associated with lower odds of higher overall ratings. For example, attending a South Asian medical school is associated with 0.32 (95% CI 0.20-0.51) greater odds of being in the bottom quartile of overall ratings relative to US or Canadian graduates. Variance inflation factor analysis suggests that there does not exist a problematic amount of collinearity, and all variables have a variance inflation factor below 5. Additionally, we do not find any strong influential outliers on binned residual analysis. We find similar results when using Face++'s Age predictions (Table S3 in Multimedia Appendix 3) and when treating age and facial attractiveness as discrete variables (Table S6 in Multimedia Appendix 7). We present results analyzing overall ratings using cut points at the 75th percentile and the median in Table S7 in Multimedia Appendix 8.

**Table 2:** Multivariate logistic regression analysis of factors associated with primary care physicians' overall, bedside manner, and wait time ratings on Zocdoc.

Characteristics		Overall^a^, OR^b^ (95% CI)	Bedside manner^c^, OR (95% CI)	Wait time^d^, OR (95% CI)
**Gender**				
	Female	Reference level	Reference level	Reference level
	Male	1.38 (0.97-1.97)	1.25 (0.88-1.79)	1.13 (0.79-1.61)
**Age quartile**				
	Q1 (0-25)	Reference level	Reference level	Reference level
	Q2 (25-30)	1.12 (0.76-1.64)	0.99 (0.67-1.45)	0.99 (0.67-1.47)
	Q3 (30-37)	0.69 (0.46-1.04)	0.66 (0.44-0.99)^e^	0.71 (0.46-1.07)
	Q4 (37-65)	0.49 (0.30-0.81)^f^	0.51 (0.31-0.84)^f^	0.68 (0.41-1.12)
**Race**				
	White	Reference level	Reference level	Reference level
	Asian	1.14 (0.82-1.59)	1.22 (0.88-1.70)	1.12 (0.80-1.57)
	Black	0.98 (0.58-1.69)	1.08 (0.64-1.88)	0.67 (0.41-1.12)
	Hispanic	0.88 (0.57-1.38)	0.94 (0.61-1.47)	1.22 (0.77-1.99)
**Facial attractiveness quartile**				
	Q1 (0-47)	Reference level	Reference level	Reference level
	Q2 (47-55)	0.81 (0.57-1.15)	0.87 (0.61-1.23)	0.87 (0.61-1.23)
	Q3 (55-64)	0.98 (0.68-1.42)	0.98 (0.67-1.42)	1.07 (0.73-1.55)
	Q4 (64-90)	1.09 (0.73-1.62)	0.87 (0.59-1.29)	1.18 (0.79-1.77)
Top 30 Ranking		0.68 (0.42-1.11)	0.56 (0.35-0.91)^e^	0.87 (0.53-1.43)
**Region**				
	United States or Canada	Reference level	Reference level	Reference level
	Africa	0.96 (0.39-2.55)	0.76 (0.31-1.94)	1.48 (0.60-4.00)
	Caribbean	0.93 (0.59-1.51)	0.93 (0.59-1.51)	1.78 (1.06, 3.10)^e^
	East or Southeast Asia	0.83 (0.37-1.95)	1.02 (0.45-2.50)	0.78 (0.36-1.76)
	Europe	1.01 (0.54-1.97)	0.83 (0.45-1.58)	0.73 (0.40-1.36)
	Latin America	0.56 (0.31-1.02)	0.63 (0.34-1.19)	0.73 (0.40-1.37)
	Middle East	0.69 (0.32-1.58)	0.74 (0.34-1.68)	Reference level
	Other	0.80 (0.07-17.7)	0.19 (0.01-2.03)	0.87 (0.08-19.3)
	South Asia	0.32 (0.20-0.51)^g^	0.30 (0.19-0.48)^g^	Reference level
**Degree**				
	DO^h^	Reference level	Reference level	Reference level
	MD^i^	0.88 (0.60-1.29)	0.79 (0.53-1.16)	0.77 (0.52-1.13)
Number of European languages		0.78 (0.63-0.96)^e^	0.77 (0.62-0.95)^e^	0.62 (0.50, 0.77)^g^
Number of East or Southeast Asian languages		0.88 (0.59-1.36)	0.77 (0.52-1.17)	0.78 (0.53-1.18)
Number of South Asian languages		1.05 (0.87-1.29)	1.02 (0.84-1.24)	1.00 (0.83-1.22)
Number of Middle Eastern languages		0.58 (0.36-0.94)^e^	0.58 (0.36-0.94)^e^	0.50 (0.30, 0.84)^f^
Number of African languages		0.73 (0.34-1.64)	0.84 (0.40-2.02)	0.71 (0.33-1.53)
Number of Creole languages		0.14 (0.01-1.02)	Reference level	0.21 (0.01-1.57)

^a^Akaike information criterion (AIC)=1606; Bayesian information criterion (BIC)=1749; deviance=1552; and area under the receiver operating characteristic curve (AUROC)=0.654.

^b^OR: odds ratio.

^c^AIC=1597; BIC=1739; deviance=1543; and AUROC=0.655.

^d^AIC=1542; BIC=1684; deviance=1488; and AUROC=0.666.

^e^*P*<.05.

^f^*P*<.01.

^g^*P*<.001.

^h^DO: doctor of osteopathic medicine.

^i^MD: doctor of medicine.

## Discussion

This study aimed to examine the impact of physician characteristics, specifically gender, age, facial attractiveness, medical school ranking, foreign degree status, and number of languages spoken, on patients' ratings of primary care physicians on Zocdoc, a platform that only publishes reviews from verified patients. Our univariate findings show that older age, lower facial attractiveness, foreign degrees, allopathic degrees, and more languages spoken in office are negatively associated with the overall rating of primary care doctors. However, the effect sizes of these factors are small and may not be clinically significant. For example, we find a 0.14 gap in median overall rating between doctors with a US degree and doctors with a Latin American degree. Prior research identifies that a half-star improvement in ratings leads to a 10% increase in likelihood that a physician will fill an appointment [11]. A back-of-the-envelope calculation suggests that this 0.14 gap is equivalent to a 2.8% increase in appointments.

To our knowledge, this paper is the first to examine the association between the number of languages, age, facial attractiveness, degree type, foreign graduate status, school ranking, and race with web-based physician reviews, and the first to examine the association between the number of languages and school ranking on patient satisfaction in general.

Our findings on the role of degree type and school ranking are generally consistent with past research. For example, a national telephone survey found that patients of osteopathic physicians generally reported higher rates of satisfaction than patients of allopathic physicians [49]. Past research has found that osteopathic physicians are more likely than allopathic physicians to call patients by their first name, provide information on the underlying causes of their illnesses, and have conversations with them about the social, family, and emotional implications of their medical conditions, all of which may contribute to higher satisfaction rates [50]. In addition, past research finds little correlation between medical school ranking and patient mortality and readmission rates, suggesting an elite ranking has negligible impact on patient care [51].

While it may be easy to hypothesize reasons for differences based on age, facial attractiveness, or foreign degree status, it is not entirely clear what could drive the differences we observe with the number of languages. We find that more languages spoken is associated with a decrease in rating. The number of languages may serve as a proxy for foreign-born status, but we cannot be certain. It is possible that physicians who speak 2 languages are more likely to be bilingual US natives whereas those who speak more than 2 languages are more likely to be immigrants. Survey data have found that patients report lower satisfaction with international medical graduates [25,52]. Older Medicare patients notably have lower mortality rates when treated by international graduates compared to US graduates [53].

In addition, we did not find any numerical rating differences by race. This is inconsistent with past work on findings on a direct-to-consumer telemedicine platform in which patients report lower satisfaction with Asian and Black physicians [25]. Moreover, we did not find any numerical rating differences by gender, which is inconsistent with some previous papers on the topic [18,19,21,22,54]. Zocdoc, however, differs from many other physician review platforms in that reviews can only come from patients after they have received care from their respective physician [33]. On third-party independent platforms like RateMDs, Google Reviews, or Healthgrades, anyone may post a review regardless of whether they have actually seen the provider. Additionally, Zocdoc solicits reviews after each appointment which may counter the typical biases of only extremely satisfied or unsatisfied consumers leaving reviews, leading to larger, more representative samples. Lastly, Zocdoc only publishes patient reviews that do not violate their community standards barring those that include personal information, pricing specifics, profanity, claims about the accuracy of a provider’s treatment or diagnosis, or promotional content.

Several limitations must be considered in interpreting the study results. First, our study only examined patient reviews of physicians on one web-based review platform and results may not generalize to other platforms. For instance, millennial women, New Yorkers, and residents of urban areas are disproportionately represented as patients on Zocdoc [55]. Moreover, while we find no differences by gender, female physicians on review platforms such as RateMDs and Google Reviews have been found to have lower numerical ratings than men [20-22]. This may be driven by a lack of verification in review postings or a lack of review moderation. Because Zocdoc moderates reviews, we cannot determine how results might change if reviews that violated community standards were included in the analysis. For example, it is possible that immigrants or women are more likely to receive lower but also more profane reviews. If Zocdoc removes these profane reviews, any disparities we are able to observe may be attenuated. In addition, because most physicians have a very high mean rating, there may be a ceiling effect that makes it difficult to discern relationships between ratings and physician factors. Second, we restrict the study to primary care physicians and the results may differ for other specialties. For example, one small study of 271 sports medicine surgeons found gender differences in ratings on 1 of 3 platform studies [23]. Third, we limit our analysis to differences in numerical ratings. However, linguistic analyses may yield different types of bias; research on Zocdoc does find that text reviews of women physicians are more informal and emotional than reviews of men [19]. Fourth, we rely on automated face classification software to classify facial attractiveness, race, and age. However, while past social science work may have relied on human raters to code unstructured information, many social scientists have fully moved toward using automated algorithmic procedures [38-43,47,48,56-59]. Moreover, while we do not have access to the ground truth data on race and age, patients most likely do not have access to this information and instead are influenced by perceived race and age. Fifth, the number of languages spoken in a provider’s office may correspond to the number of languages spoken by the physicians themselves or by their staff, and what this variable proxies is unclear. Sixth, the overall explanatory power of our multivariable models is fair and may be limited due to the many factors that play key roles in patient satisfaction that we are unable to observe. Seventh, our data do not permit any mechanistic or causal interpretations. Despite these limitations, our study represents an important step in understanding the potential biases in web-based doctor reviews and highlights the need for further research in this area.

Although our study suggests that physician factors have a real but limited impact on numerical ratings, it is important to note that bias may play out in many forms. For example, a physician's appearance or accent may impact a patient's trust, confidence, or satisfaction with their physician, which could in turn influence their take-up of preventative services and lead to either better or worse health outcomes [60]. Such a phenomenon would not be captured in our analysis of numerical ratings, but our results open the door to investigating such phenomenon across facial attractiveness, multilingualism, education, and age more deeply. In conclusion, this study provides insights into the association between physician characteristics and patients' web-based ratings of primary care physicians. Future research should consider textual analyses of reviews, investigate how factors like facial attractiveness interact with patient outcomes, and explore whether the findings of this study generalize to other medical specialties, review platforms, or patient populations. Ultimately, our findings underscore the need for greater awareness of potential biases in web-based doctor reviews and the importance of considering a range of factors in evaluating health care providers.

## Appendix

Multimedia Appendix 1 Languages.

Multimedia Appendix 2 Comparison of Kairos and Face++ on Age.

Multimedia Appendix 3 Multivariate Results using Face++ Age Predictions.

Multimedia Appendix 4 Locations of Primary Care Doctors.

Multimedia Appendix 5 Median Outcome by Physician Demographic Factor.

Multimedia Appendix 6 Higher resolution version of Figure 1. Overall primary care physician Zocdoc ratings by physician demographic factor.

Multimedia Appendix 7 Multivariate Logistic Regression Results with Discrete Age and Facial Attractiveness.

Multimedia Appendix 8 Multivariate Logistic Regression Results with Alternate Overall Rating Cut Point.

## Data Availability

The data sets generated during or analyzed during this study are available in the Open Science Framework repository.
