# Supplementary material for: Examining the Role of Physician Characteristics in Web-Based Verified Primary Care Physician Reviews: Observational Study
Source: J Med Internet Res. 2024 Jul 29;26:e51672. doi: 10.2196/51672 (PMC11319894; doi:10.2196/51672)
Supplement: Multimedia Appendix 1 [file jmir_v26i1e51672_app1.docx]

**Appendix Table S1. Languages**

|  | **Language** | **Number of Speakers** |
| --- | --- | --- |
| **European Languages** |  |  |
|  | English | 1455 |
|  | Spanish | 278 |
|  | Russian | 36 |
|  | French | 32 |
|  | Armenian | 11 |
|  | Greek | 10 |
|  | Romanian | 9 |
|  | Italian | 8 |
|  | Polish | 7 |
|  | Ukrainian | 7 |
|  | German | 3 |
|  | Serbo-Croatian | 3 |
|  | Hungarian | 3 |
|  | Georgian | 2 |
|  | Portuguese | 2 |
|  | Bosnian | 2 |
|  | Dutch | 2 |
|  | Serbian | 2 |
|  | Czech | 1 |
|  | Albanian | 1 |
|  | Yiddish | 1 |
|  | Croatian | 1 |
|  | Swedish | 1 |
|  | Macedonian | 1 |
| **East and Southeast Asian Languages** |  |  |
|  | Chinese (Mandarin) | 30 |
|  | Vietnamese | 23 |
|  | Chinese (Cantonese) | 17 |
|  | Tagalog | 13 |
|  | Korean | 8 |
|  | Japanese | 3 |
|  | Taiwanese | 2 |
|  | Chinese (Fujian) | 2 |
|  | Burmese | 2 |
|  | Filipino | 1 |
|  | Cebuano | 1 |
|  | Chinese (Wenzhounese) | 1 |
|  | Thai | 1 |
|  | Lao | 1 |
| **South Asian Languages** |  |  |
|  | Hindi | 142 |
|  | Urdu | 86 |
|  | Punjabi | 37 |
|  | Gujarati | 32 |
|  | Telugu | 20 |
|  | Bengali | 15 |
|  | Tamil | 14 |
|  | Malayalam | 13 |
|  | Kannada | 6 |
|  | Marathi | 4 |
|  | Nepali | 3 |
|  | Sinhalese | 1 |
| **Middle Eastern Languages** |  |  |
|  | Arabic | 50 |
|  | Farsi | 34 |
|  | Turkish | 2 |
|  | Hebrew | 8 |
| **African Languages** |  |  |
|  | Igbo | 6 |
|  | Yoruba | 5 |
|  | Amharic | 3 |
|  | Swahili | 2 |
|  | Tigrinya | 2 |
|  | Oromo | 1 |
|  | Xhosa | 1 |
|  | Akan | 1 |
|  | Zulu | 1 |
|  | Afrikaans | 1 |
| **Creole Languages** |  |  |
|  | Creole (Haitian) | 3 |
|  | Creole | 2 |
