# Supplementary material for: Examining the Role of Physician Characteristics in Web-Based Verified Primary Care Physician Reviews: Observational Study
Source: J Med Internet Res. 2024 Jul 29;26:e51672. doi: 10.2196/51672 (PMC11319894; doi:10.2196/51672)
Supplement: Multimedia Appendix 2 [file jmir_v26i1e51672_app2.docx]

**Appendix Table S2. Comparison of Kairos and Face++ on Age**

|  | **Kairos Age** | | | | **Face++ Age** | | | |
| --- | --- | --- | --- | --- | --- | --- | --- | --- |
| **Characteristic** | **25th, N = 409** | **50th, N = 336** | **75th, N = 392** | **>75th, N = 316** | **25th, N = 366** | **50th, N = 384** | **75th, N = 362** | **>75th, N = 340** |
| Overall Rating | 4.83 (4.69, 4.92) | 4.83 (4.70, 4.92) | 4.82 (4.67, 4.93) | 4.78 (4.61, 4.93) | 4.85 (4.73, 4.94) | 4.84 (4.69, 4.93) | 4.79 (4.66, 4.90) | 4.78 (4.61, 4.93) |
| Bedside Manner | 4.89 (4.77, 4.97) | 4.89 (4.78, 4.99) | 4.88 (4.73, 4.99) | 4.84 (4.70, 4.96) | 4.91 (4.80, 5.00) | 4.90 (4.78, 4.99) | 4.86 (4.73, 4.95) | 4.83 (4.67, 4.96) |
| Missing | 5 | 14 | 10 | 6 | 11 | 9 | 9 | 6 |
| Wait Time | 4.72 (4.50, 4.86) | 4.67 (4.49, 4.83) | 4.65 (4.41, 4.83) | 4.62 (4.38, 4.79) | 4.75 (4.58, 4.88) | 4.70 (4.50, 4.82) | 4.63 (4.40, 4.81) | 4.58 (4.35, 4.79) |
| Missing | 5 | 14 | 10 | 6 | 11 | 9 | 9 | 6 |
| Number of Reviews | 54 (22, 239) | 53 (21, 185) | 58 (18, 235) | 63 (16, 292) | 46 (15, 186) | 64 (22, 284) | 82 (30, 292) | 43 (15, 181) |
| Median, (IQR) | | | | | | | | |
