# Supplementary material for: Examining the Role of Physician Characteristics in Web-Based Verified Primary Care Physician Reviews: Observational Study
Source: J Med Internet Res. 2024 Jul 29;26:e51672. doi: 10.2196/51672 (PMC11319894; doi:10.2196/51672)
Supplement: Multimedia Appendix 3 [file jmir_v26i1e51672_app3.docx]

**Appendix Table S3. Multivariate Results using Face++ Age Predictions**

|  | **Overall** | **Bedside Manner** | **Wait Time** |
| --- | --- | --- | --- |
| **Characteristic** | **OR** | **OR** | **OR** |
| Gender |  |  |  |
| Female | — | — | — |
| Male | 1.03 (0.80, 1.33) | 0.97 (0.75, 1.25) | 1.02 (0.78, 1.33) |
| Age Quartile |  |  |  |
| Q1 (0-34] | — | — | — |
| Q2 (34-44] | 0.78 (0.54, 1.13) | 1.00 (0.69, 1.45) | 0.81 (0.54, 1.20) |
| Q3 (44-54] | 0.72 (0.49, 1.06) | 0.69 (0.48, 1.01) | 0.49 (0.33, 0.73)*** |
| Q4 (54-87] | 0.51 (0.34, 0.78)** | 0.55 (0.36, 0.82)** | 0.44 (0.28, 0.67)*** |
| Race |  |  |  |
| White | — | — | — |
| Asian | 1.10 (0.79, 1.53) | 1.15 (0.83, 1.61) | 1.01 (0.72, 1.43) |
| Black | 0.86 (0.51, 1.49) | 0.93 (0.55, 1.62) | 0.54 (0.32, 0.92)* |
| Hispanic | 0.84 (0.55, 1.32) | 0.88 (0.57, 1.38) | 1.07 (0.67, 1.74) |
| Facial Attractiveness Quartile |  |  |  |
| Q1 (0-47] | — | — | — |
| Q2 (47-55] | 0.80 (0.57, 1.13) | 0.85 (0.60, 1.20) | 0.80 (0.57, 1.14) |
| Q3 (55-64] | 0.96 (0.66, 1.38) | 0.93 (0.64, 1.36) | 0.93 (0.63, 1.36) |
| Q4 (64-90] | 1.10 (0.74, 1.63) | 0.85 (0.57, 1.25) | 0.98 (0.66, 1.48) |
| Top 30 Ranking | 0.67 (0.42, 1.09) | 0.55 (0.35, 0.89)* | 0.85 (0.52, 1.41) |
| Region |  |  |  |
| U.S./Canada | — | — | — |
| Africa | 0.94 (0.39, 2.50) | 0.76 (0.31, 1.95) | 1.54 (0.62, 4.20) |
| Caribbean | 0.88 (0.55, 1.42) | 0.89 (0.56, 1.44) | 1.65 (0.98, 2.90) |
| East or Southeast Asia | 0.86 (0.39, 2.02) | 1.06 (0.47, 2.60) | 0.81 (0.37, 1.83) |
| Europe | 1.00 (0.54, 1.93) | 0.84 (0.46, 1.59) | 0.79 (0.44, 1.47) |
| Latin America | 0.55 (0.31, 1.02) | 0.64 (0.35, 1.20) | 0.77 (0.42, 1.45) |
| Middle East | 0.70 (0.32, 1.61) | 0.78 (0.36, 1.78) | 5.10 (1.74, 19.1)** |
| Other | 0.68 (0.06, 15.1) | 0.16 (0.01, 1.82) | 0.94 (0.08, 21.6) |
| South Asia | 0.33 (0.21, 0.53)*** | 0.31 (0.19, 0.49)*** | 0.53 (0.33, 0.85)** |
| Degree |  |  |  |
| D.O. | — | — | — |
| M.D. | 0.93 (0.63, 1.36) | 0.83 (0.56, 1.22) | 0.83 (0.56, 1.21) |
| Number of European Languages | 0.77 (0.62, 0.96)* | 0.76 (0.61, 0.94)* | 0.62 (0.50, 0.77)*** |
| Number of East or Southeast Asian Languages | 0.90 (0.61, 1.39) | 0.79 (0.53, 1.19) | 0.80 (0.54, 1.21) |
| Number of South Asian Languages | 1.04 (0.86, 1.28) | 1.01 (0.84, 1.23) | 0.99 (0.82, 1.21) |
| Number of Middle Eastern Languages | 0.56 (0.35, 0.90)* | 0.56 (0.35, 0.90)* | 0.49 (0.29, 0.82)** |
| Number of African Languages | 0.72 (0.34, 1.61) | 0.87 (0.41, 2.08) | 0.76 (0.36, 1.66) |
| Number of Creole Languages | 0.14 (0.01, 0.98) | NA | 0.19 (0.01, 1.41) |
| AIC | 1609 | 1595 | 1526 |
| BIC | 1752 | 1736 | 1667 |
| Deviance | 1555 | 1541 | 1472 |
| AUROC | 0.650 | 0.660 | 0.680 |
| *p<0.05; **p<0.01; ***p<0.001 | | | |
| OR = Odds Ratio | | | |
