# Supplementary material for: Examining the Role of Physician Characteristics in Web-Based Verified Primary Care Physician Reviews: Observational Study
Source: J Med Internet Res. 2024 Jul 29;26:e51672. doi: 10.2196/51672 (PMC11319894; doi:10.2196/51672)
Supplement: Multimedia Appendix 4 [file jmir_v26i1e51672_app4.docx]

**Appendix Table S4. Locations of Primary Care Doctors**

| **City** | **n (%)** | **% of U.S. population** |
| --- | --- | --- |
| Houston | 249 (17%) | 0.69% |
| Chicago | 246 (17%) | 0.81% |
| New York City | 222 (15%) | 2.55% |
| Los Angeles | 119 (8.2%) | 1.16% |
| Phoenix | 88 (6.0%) | 0.49% |
| Baltimore | 76 (5.2%) | 0.17% |
| Detroit | 63 (4.3%) | 0.19% |
| Denver | 60 (4.1%) | 0.21% |
| Washington, D.C. | 54 (3.7%) | 0.20% |
| Fort Worth | 49 (3.4%) | 0.28% |
| Philadelphia | 47 (3.2%) | 0.47% |
| Dallas | 35 (2.4%) | 0.39% |
| San Antonio | 25 (1.7%) | 0.44% |
| San Francisco | 20 (1.4%) | 0.25% |
| Austin | 18 (1.2%) | 0.29% |
| Boston | 14 (1.0%) | 0.20% |
| Jacksonville | 13 (0.9%) | 0.29% |
| Charlotte | 9 (0.6%) | 0.26% |
| El Paso | 9 (0.6%) | 0.20% |
| San Jose | 9 (0.6%) | 0.30% |
| Seattle | 9 (0.6%) | 0.22% |
| Las Vegas | 6 (0.4%) | 0.19% |
| Indianapolis | 5 (0.3%) | 0.27% |
| Louisville | 3 (0.2%) | 0.19% |
| Nashville | 2 (0.1%) | 0.20% |
| Portland | 2 (0.1%) | 0.19% |
| San Diego | 2 (0.1%) | 0.42% |
| Columbus | 1 (<0.1%) | 0.27% |
| Memphis | 0 (0%) | 0.19% |
| Oklahoma City | 0 (0%) | 0.21% |
