# Supplementary material for: Examining the Role of Physician Characteristics in Web-Based Verified Primary Care Physician Reviews: Observational Study
Source: J Med Internet Res. 2024 Jul 29;26:e51672. doi: 10.2196/51672 (PMC11319894; doi:10.2196/51672)
Supplement: Multimedia Appendix 5 [file jmir_v26i1e51672_app5.docx]

**Appendix Table S5. Median Outcome by Physician Demographic Factor**

|  | **Gender** | | **Age Quartile** | | | | **Race** | | | | **Facial Attractiveness Quartile** | | | |
| --- | --- | --- | --- | --- | --- | --- | --- | --- | --- | --- | --- | --- | --- | --- |
| **Characteristic** | **Female, N = 675** | **Male, N = 780** | **Q1, N = 409** | **Q2, N = 336** | **Q3, N = 392** | **Q4, N = 316** | **Asian, N = 598** | **Black, N = 107** | **Hispanic, N = 149** | **White, N = 600** | **Q1, N = 363** | **Q2, N = 363** | **Q3, N = 363** | **Q4, N = 363** |
| Overall Rating | 4.83 (4.68, 4.92) | 4.82 (4.67, 4.94) | 4.83 (4.69, 4.92) | 4.83 (4.70, 4.92) | 4.82 (4.67, 4.93) | 4.78 (4.61, 4.93) | 4.82 (4.67, 4.93) | 4.83 (4.67, 4.92) | 4.84 (4.66, 4.92) | 4.82 (4.68, 4.93) | 4.81 (4.66, 4.93) | 4.82 (4.63, 4.92) | 4.82 (4.68, 4.92) | 4.84 (4.72, 4.93) |
| Bedside Manner | 4.89 (4.75, 4.97) | 4.87 (4.74, 4.99) | 4.89 (4.77, 4.97) | 4.89 (4.78, 4.99) | 4.88 (4.73, 4.99) | 4.84 (4.70, 4.96) | 4.87 (4.75, 4.97) | 4.89 (4.75, 4.96) | 4.90 (4.72, 5.00) | 4.88 (4.75, 4.98) | 4.86 (4.74, 4.98) | 4.87 (4.71, 4.97) | 4.89 (4.75, 4.99) | 4.90 (4.77, 4.98) |
| Missing | 12 | 23 | 5 | 14 | 10 | 6 | 19 | 0 | 2 | 14 | 5 | 11 | 12 | 7 |
| Wait Time | 4.67 (4.45, 4.83) | 4.66 (4.43, 4.83) | 4.72 (4.50, 4.86) | 4.67 (4.49, 4.83) | 4.65 (4.41, 4.83) | 4.62 (4.38, 4.79) | 4.66 (4.45, 4.83) | 4.60 (4.36, 4.78) | 4.71 (4.50, 4.82) | 4.68 (4.45, 4.83) | 4.64 (4.40, 4.81) | 4.63 (4.40, 4.82) | 4.70 (4.46, 4.85) | 4.71 (4.53, 4.85) |
| Missing | 12 | 23 | 5 | 14 | 10 | 6 | 19 | 0 | 2 | 14 | 5 | 11 | 12 | 7 |
| Number of Reviews | 56 (22, 206) | 58 (17, 253) | 54 (22, 239) | 53 (21, 185) | 58 (18, 235) | 63 (16, 292) | 61 (21, 251) | 76 (34, 224) | 55 (21, 178) | 49 (16, 224) | 50 (18, 174) | 53 (17, 262) | 54 (18, 204) | 73 (24, 313) |
| Median (IQR) | | | | | | | | | | | | | | |

|  | **Top 30** | | **Degree** | | **Medical School Location** | | | | | | | | |
| --- | --- | --- | --- | --- | --- | --- | --- | --- | --- | --- | --- | --- | --- |
| **Characteristic** | **>30, N = 1,348** | **Top 30, N = 106** | **D.O., N = 279** | **M.D., N = 1,176** | **Africa, N = 28** | **Caribbean, N = 141** | **East Asia, N = 40** | **Europe, N = 68** | **Latin America, N = 59** | **Middle East, N = 38** | **Other, N = 3** | **South Asia, N = 180** | **US/Canada,  N = 897** |
| Overall Rating | 4.82 (4.67, 4.93) | 4.81 (4.66, 4.91) | 4.86 (4.74, 4.96) | 4.81 (4.66, 4.92) | 4.80 (4.67, 4.88) | 4.84 (4.73, 4.93) | 4.82 (4.68, 4.92) | 4.79 (4.64, 4.86) | 4.71 (4.54, 4.88) | 4.78 (4.64, 4.92) | 4.80 (4.67, 4.83) | 4.73 (4.54, 4.84) | 4.85 (4.70, 4.94) |
| Bedside Manner | 4.88 (4.75, 4.98) | 4.85 (4.67, 4.96) | 4.91 (4.81, 5.00) | 4.87 (4.73, 4.97) | 4.86 (4.73, 4.92) | 4.90 (4.79, 5.00) | 4.89 (4.78, 4.96) | 4.86 (4.70, 4.91) | 4.83 (4.59, 4.97) | 4.84 (4.69, 4.96) | 4.69 (4.61, 4.73) | 4.80 (4.58, 4.90) | 4.90 (4.77, 5.00) |
| Missing | 31 | 4 | 4 | 31 | 0 | 4 | 1 | 2 | 2 | 0 | 0 | 4 | 22 |
| Wait Time | 4.67 (4.45, 4.83) | 4.64 (4.41, 4.80) | 4.74 (4.54, 4.88) | 4.65 (4.42, 4.82) | 4.62 (4.37, 4.76) | 4.75 (4.58, 4.89) | 4.75 (4.40, 4.88) | 4.55 (4.30, 4.76) | 4.56 (4.25, 4.76) | 4.64 (4.51, 4.82) | 4.62 (4.45, 4.74) | 4.55 (4.35, 4.71) | 4.69 (4.50, 4.84) |
| Missing | 31 | 4 | 4 | 31 | 0 | 4 | 1 | 2 | 2 | 0 | 0 | 4 | 22 |
| Number of Reviews | 58 (18, 232) | 52 (33, 218) | 44 (13, 158) | 61 (20, 248) | 278 (72, 555) | 49 (13, 180) | 62 (13, 220) | 139 (36, 503) | 41 (16, 146) | 128 (40, 442) | 50 (47, 494) | 68 (16, 300) | 51 (19, 199) |
| Median (IQR) | | | | | | | | | | | | | |

|  | **European Languages** | | | **East or Southeast Asian Languages** | | | **South Asian Languages** | | | | **Middle Eastern Languages** | | **African Languages** | | **Creole Languages** | |
| --- | --- | --- | --- | --- | --- | --- | --- | --- | --- | --- | --- | --- | --- | --- | --- | --- |
| **Characteristic** | **1, N = 1,111** | **2, N = 286** | **>=3, N = 58** | **0, N = 1,373** | **1, N = 65** | **>=2, N = 17** | **0, N = 1,261** | **1, N = 70** | **2, N = 80** | **>=3, N = 44** | **0, N = 1,367** | **>=1, N = 88** | **0, N = 1,437** | **>=1, N = 18** | **0, N = 1,450** | **1, N = 5** |
| Overall Rating | 4.84 (4.69, 4.94) | 4.78 (4.62, 4.88) | 4.72 (4.54, 4.85) | 4.82 (4.67, 4.93) | 4.80 (4.68, 4.92) | 4.84 (4.68, 4.87) | 4.83 (4.68, 4.93) | 4.83 (4.68, 4.92) | 4.79 (4.59, 4.91) | 4.77 (4.56, 4.83) | 4.83 (4.68, 4.93) | 4.78 (4.60, 4.90) | 4.82 (4.67, 4.93) | 4.74 (4.62, 4.84) | 4.82 (4.67, 4.93) | 4.54 (4.46, 4.57) |
| Bedside Manner | 4.89 (4.76, 5.00) | 4.84 (4.71, 4.93) | 4.78 (4.65, 4.92) | 4.88 (4.75, 4.98) | 4.86 (4.76, 4.95) | 4.84 (4.75, 4.91) | 4.89 (4.75, 4.99) | 4.87 (4.75, 4.97) | 4.85 (4.64, 4.94) | 4.82 (4.68, 4.91) | 4.88 (4.75, 4.98) | 4.83 (4.66, 4.95) | 4.88 (4.75, 4.98) | 4.88 (4.73, 4.95) | 4.88 (4.75, 4.98) | 4.56 (4.53, 4.57) |
| Missing | 32 | 2 | 1 | 32 | 3 | 0 | 33 | 1 | 1 | 0 | 34 | 1 | 35 | 0 | 35 | 0 |
| Wait Time | 4.70 (4.50, 4.86) | 4.55 (4.33, 4.76) | 4.55 (4.30, 4.73) | 4.67 (4.45, 4.83) | 4.70 (4.40, 4.85) | 4.73 (4.32, 4.85) | 4.67 (4.45, 4.83) | 4.71 (4.50, 4.86) | 4.64 (4.47, 4.80) | 4.50 (4.31, 4.77) | 4.67 (4.45, 4.83) | 4.57 (4.36, 4.77) | 4.67 (4.45, 4.83) | 4.55 (4.32, 4.77) | 4.67 (4.45, 4.83) | 4.21 (3.95, 4.23) |
| Missing | 32 | 2 | 1 | 32 | 3 | 0 | 33 | 1 | 1 | 0 | 34 | 1 | 35 | 0 | 35 | 0 |
| Number of Reviews | 48 (16, 180) | 98 (32, 378) | 223 (60, 593) | 54 (18, 219) | 158 (34, 488) | 139 (52, 239) | 54 (19, 215) | 52 (20, 158) | 98 (30, 336) | 193 (24, 716) | 54 (18, 212) | 173 (41, 452) | 56 (19, 226) | 154 (67, 464) | 56 (19, 226) | 527 (99, 530) |
| Median (IQR) | | | | | | | | | | | | | | | | |
