# Supplementary figures and images for: Examining the Role of Physician Characteristics in Web-Based Verified Primary Care Physician Reviews: Observational Study
Source: J Med Internet Res. 2024 Jul 29;26:e51672. doi: 10.2196/51672 (PMC11319894; doi:10.2196/51672)

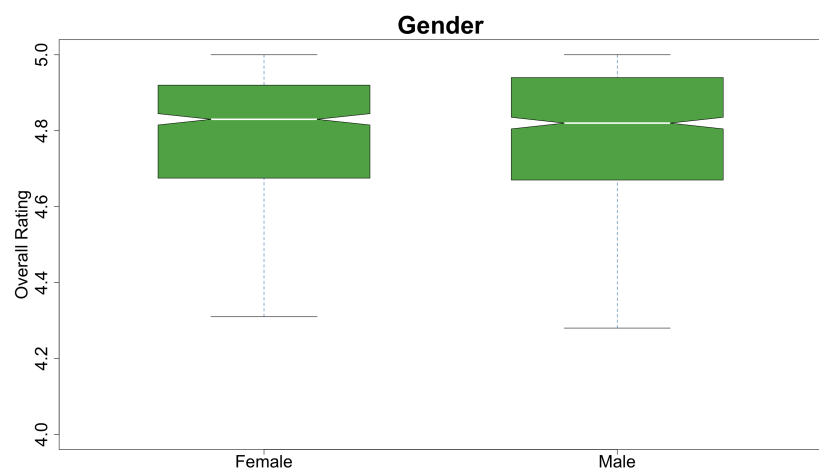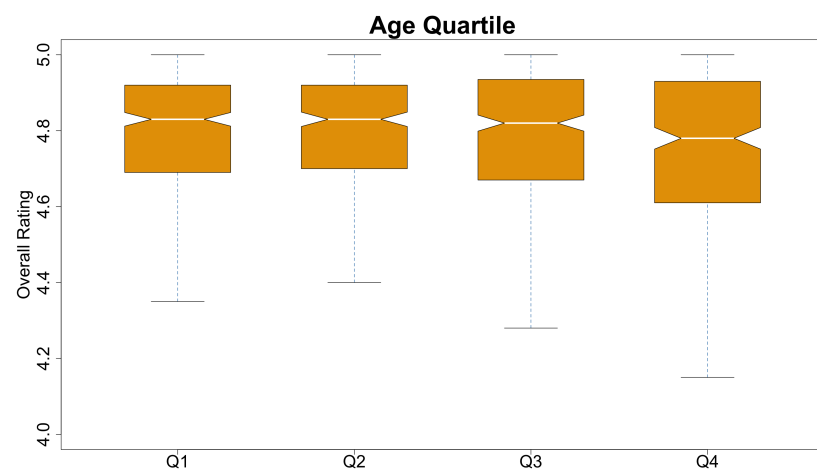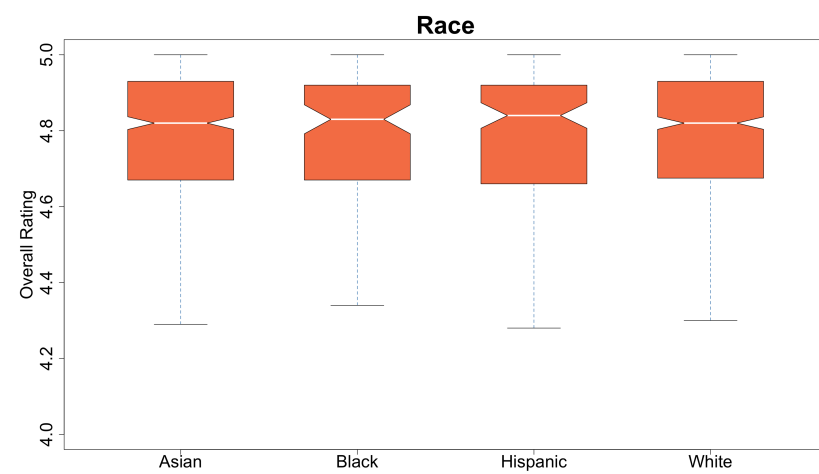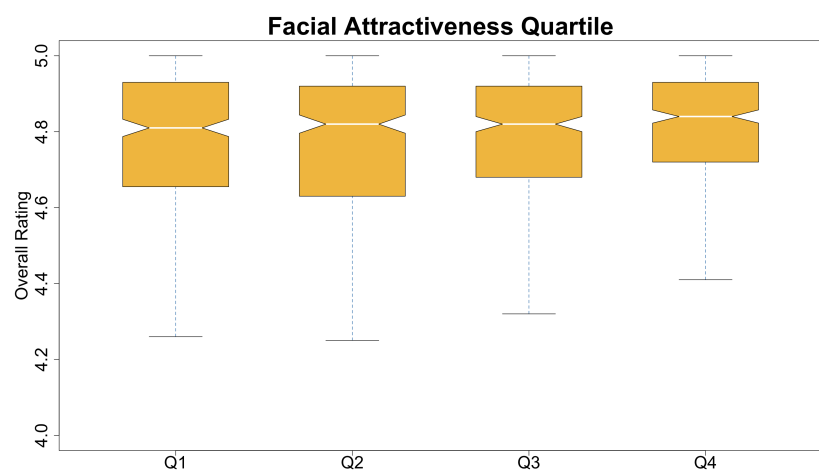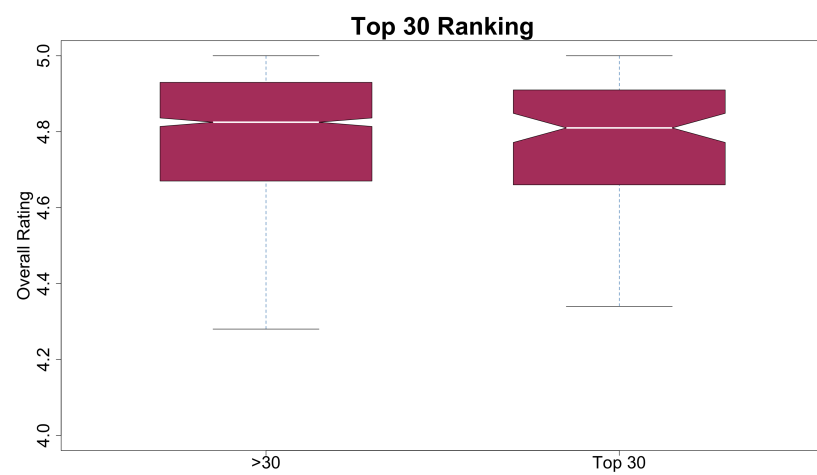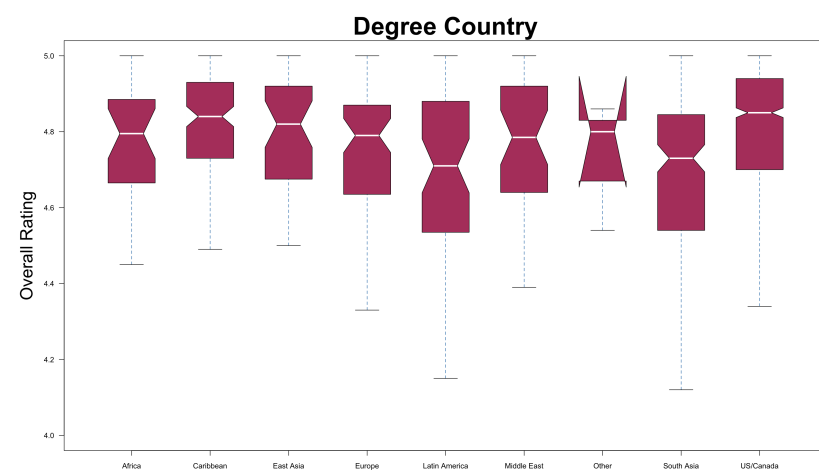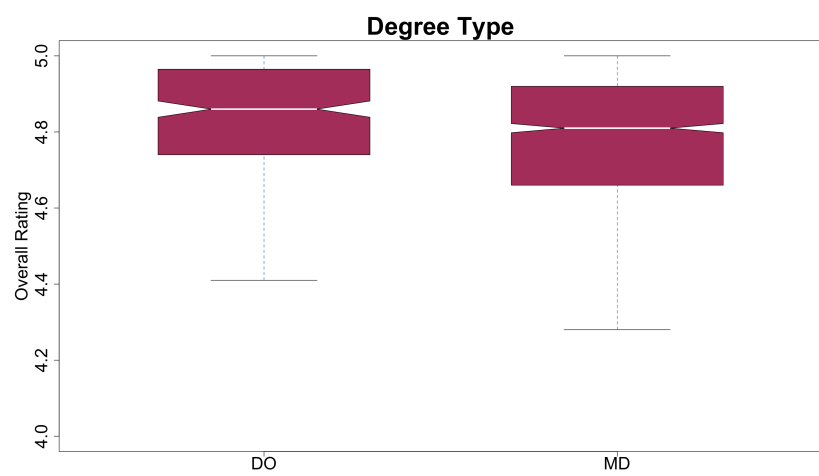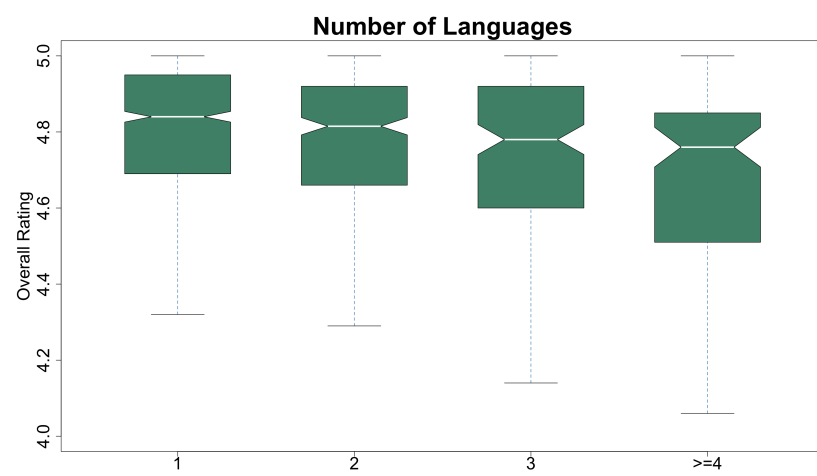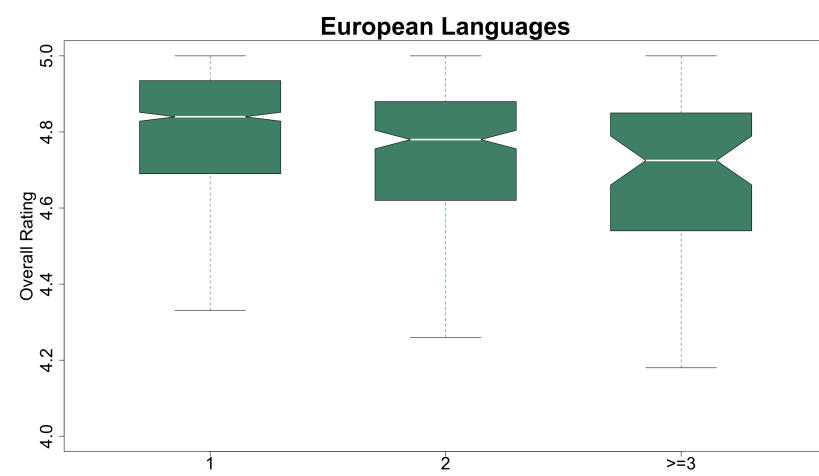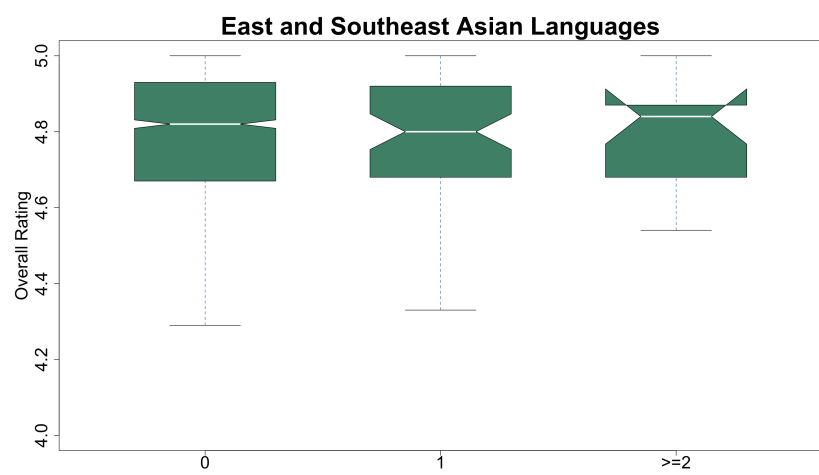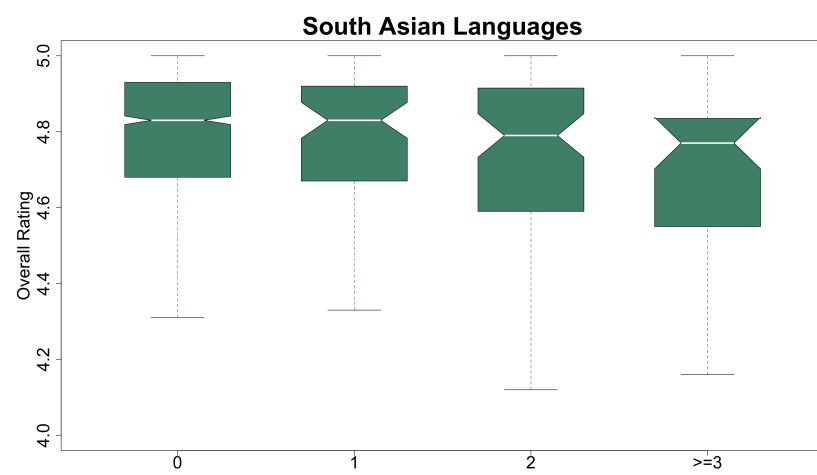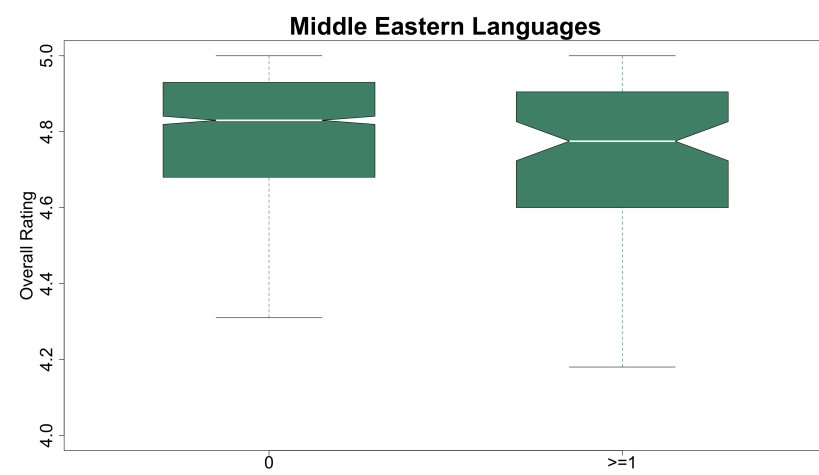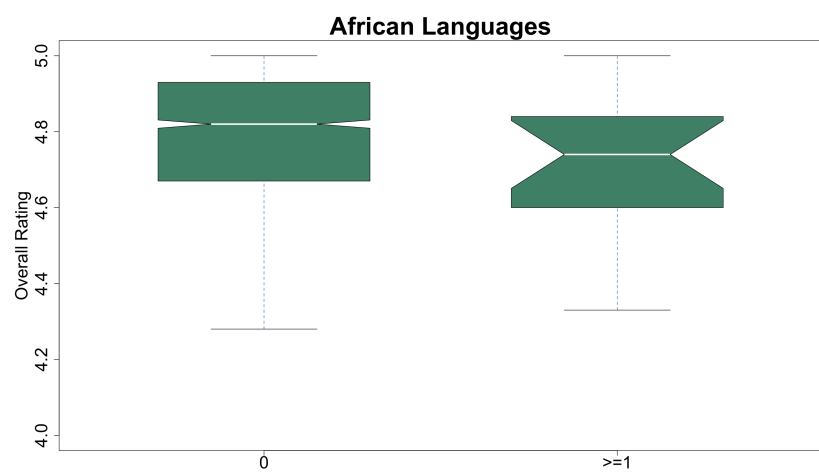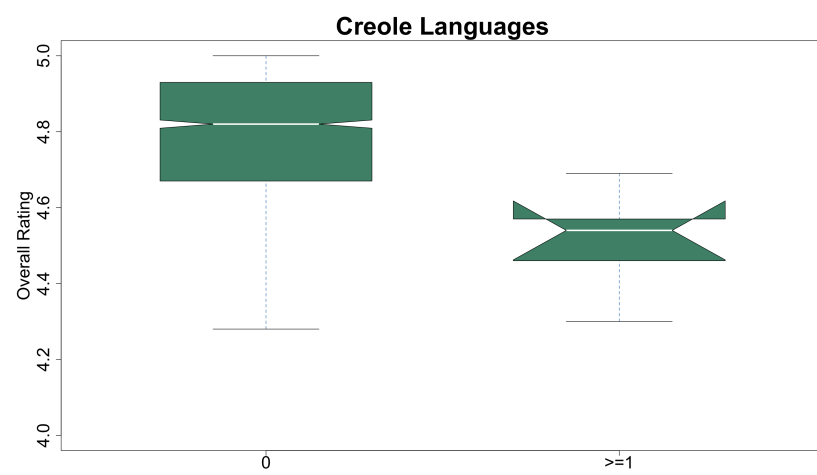

Supplement: Multimedia Appendix 6 [file jmir_v26i1e51672_app6.pdf]
