# Supplementary material for: Examining the Role of Physician Characteristics in Web-Based Verified Primary Care Physician Reviews: Observational Study
Source: J Med Internet Res. 2024 Jul 29;26:e51672. doi: 10.2196/51672 (PMC11319894; doi:10.2196/51672)
Supplement: Multimedia Appendix 7 [file jmir_v26i1e51672_app7.docx]

**Appendix Table S6. Multivariate Logistic Regression Results with Discrete Age and Facial Attractiveness**

|  | **Overall** | **Bedside Manner** | **Wait Time** |
| --- | --- | --- | --- |
| **Characteristic** | **OR** | **OR** | **OR** |
| Gender |  |  |  |
| Female | — | — | — |
| Male | 1.20 (0.86, 1.68) | 1.09 (0.78, 1.53) | 1.09 (0.77, 1.54) |
| Age | 0.98 (0.96, 1.00)* | 0.98 (0.96, 1.00)* | 0.98 (0.96, 1.00) |
| Race |  |  |  |
| White | — | — | — |
| Asian | 1.13 (0.81, 1.57) | 1.22 (0.88, 1.70) | 1.10 (0.79, 1.55) |
| Black | 1.02 (0.60, 1.76) | 1.08 (0.64, 1.87) | 0.67 (0.41, 1.12) |
| Hispanic | 0.88 (0.57, 1.36) | 0.95 (0.62, 1.47) | 1.20 (0.75, 1.94) |
| Facial Attractiveness | 1.01 (1.00, 1.02) | 1.00 (0.99, 1.01) | 1.01 (1.00, 1.02) |
| Top 30 Ranking | 0.68 (0.42, 1.11) | 0.56 (0.35, 0.91)* | 0.86 (0.53, 1.43) |
| Region |  |  |  |
| U.S./Canada | — | — | — |
| Africa | 0.98 (0.40, 2.59) | 0.77 (0.32, 1.97) | 1.53 (0.62, 4.12) |
| Caribbean | 0.91 (0.58, 1.48) | 0.92 (0.58, 1.48) | 1.74 (1.04, 3.04)* |
| East and Southeast Asia | 0.82 (0.37, 1.93) | 1.01 (0.45, 2.47) | 0.76 (0.35, 1.71) |
| Europe | 1.00 (0.54, 1.92) | 0.82 (0.44, 1.54) | 0.75 (0.41, 1.38) |
| Latin America | 0.54 (0.30, 0.99)* | 0.61 (0.33, 1.14) | 0.72 (0.39, 1.36) |
| Middle East | 0.64 (0.29, 1.46) | 0.71 (0.33, 1.61) | 4.38 (1.50, 16.4)* |
| Other | 0.66 (0.06, 14.5) | 0.17 (0.01, 1.81) | 0.78 (0.07, 17.2) |
| South Asia | 0.32 (0.20, 0.50)*** | 0.30 (0.19, 0.48)*** | 0.47 (0.30, 0.76)** |
| Degree |  |  |  |
| D.O. | — | — | — |
| M.D. | 0.90 (0.61, 1.32) | 0.80 (0.54, 1.17) | 0.79 (0.53, 1.15) |
| Number of European Languages | 0.77 (0.62, 0.95)* | 0.76 (0.61, 0.94)* | 0.62 (0.49, 0.77)*** |
| Number of East or Southeast Asian Languages | 0.88 (0.59, 1.36) | 0.77 (0.52, 1.17) | 0.77 (0.53, 1.17) |
| Number of South Asian Languages | 1.06 (0.88, 1.29) | 1.02 (0.85, 1.24) | 1.01 (0.83, 1.23) |
| Number of Middle Eastern Languages | 0.58 (0.36, 0.93)* | 0.58 (0.36, 0.93)* | 0.50 (0.30, 0.84)** |
| Number of African Languages | 0.75 (0.36, 1.68) | 0.86 (0.41, 2.06) | 0.72 (0.34, 1.56) |
| Number of Creole Languages | 0.14 (0.01, 1.00) | NA | 0.20 (0.01, 1.47) |
| AIC | 1608 | 1596 | 1538 |
| BIC | 1729 | 1717 | 1659 |
| Deviance | 1562 | 1550 | 1492 |
| AUROC | 0.647 | 0.647 | 0.665 |
| *p<0.05; **p<0.01; ***p<0.001 | | | |
| OR = Odds Ratio | | | |
