# Supplementary material for: Examining the Role of Physician Characteristics in Web-Based Verified Primary Care Physician Reviews: Observational Study
Source: J Med Internet Res. 2024 Jul 29;26:e51672. doi: 10.2196/51672 (PMC11319894; doi:10.2196/51672)
Supplement: Multimedia Appendix 8 [file jmir_v26i1e51672_app8.docx]

**Appendix Table S7. Multivariate Logistic Regression Results with Alternate Overall Rating Cut Point**

|  | **Above 75th percentile** | **Above median** |
| --- | --- | --- |
| **Characteristic** | **OR** | **OR** |
| Gender |  |  |
| Female | — | — |
| Male | 1.42 (1.00, 2.01) | 1.04 (0.77, 1.41) |
| Age Quartile |  |  |
| Q1 (0-34] | — | — |
| Q2 (34-44] | 0.91 (0.62, 1.33) | 1.06 (0.77, 1.45) |
| Q3 (44-54] | 1.01 (0.67, 1.53) | 0.93 (0.65, 1.33) |
| Q4 (54-87] | 0.90 (0.54, 1.49) | 0.83 (0.54, 1.28) |
| Race |  |  |
| White | — | — |
| Asian | 0.91 (0.62, 1.33) | 1.06 (0.77, 1.45) |
| Black | 1.01 (0.67, 1.53) | 0.93 (0.65, 1.33) |
| Hispanic | 0.90 (0.54, 1.49) | 0.83 (0.54, 1.28) |
| Facial Attractiveness Quartile |  |  |
| Q1 (0-47] | — | — |
| Q2 (47-55] | 0.82 (0.57, 1.18) | 1.07 (0.78, 1.45) |
| Q3 (55-64] | 0.80 (0.55, 1.16) | 1.06 (0.77, 1.46) |
| Q4 (64-90] | 0.84 (0.57, 1.23) | 1.13 (0.81, 1.59) |
| Top 30 Ranking | 0.79 (0.47, 1.29) | 0.71 (0.46, 1.08) |
| Region |  |  |
| U.S./Canada | — | — |
| Africa | 0.25 (0.04, 0.93) | 0.57 (0.23, 1.34) |
| Caribbean | 0.89 (0.56, 1.39) | 1.01 (0.69, 1.50) |
| East or Southeast Asia | 0.86 (0.34, 2.00) | 0.90 (0.44, 1.82) |
| Europe | 0.59 (0.26, 1.20) | 0.72 (0.41, 1.26) |
| Latin America | 0.75 (0.34, 1.52) | 0.77 (0.43, 1.38) |
| Middle East | 0.93 (0.36, 2.22) | 0.79 (0.37, 1.69) |
| Other | NA | 0.57 (0.03, 6.26) |
| South Asia | 0.41 (0.23, 0.70)** | 0.29 (0.19, 0.45)*** |
| Degree |  |  |
| D.O. | — | — |
| M.D. | 0.85 (0.61, 1.19) | 0.86 (0.63, 1.16) |
| Number of European Languages | 0.73 (0.55, 0.95)* | 0.64 (0.52, 0.80)*** |
| Number of East or Southeast Asian Languages | 0.53 (0.30, 0.87)* | 0.83 (0.58, 1.18) |
| Number of South Asian Languages | 0.93 (0.73, 1.15) | 1.07 (0.90, 1.28) |
